# Machine Learning for Predicting Critical Postoperative Interventions: Proof-of-Concept Study Using the INSPIRE Dataset

**DOI:** 10.2196/65327

**Published:** 2026-07-28

**Authors:** Manan Shukla, Paul Fodor, Suresh Yelika, Nicholas Ahn

**Affiliations:** 1 Renaissance School of Medicine, Stonybrook University Hospital, 118 Leeward Lane, Port Jefferson, NY, 11777, United States, 1 561-720-8383; 2 Department of Computer Science, Stony Brook University, Stony Brook, NY, United States; 3 Colorectal Surgery, Stony Brook Medicine, Stony Brook, NY, United States

**Keywords:** postoperative complication prediction, surgical risk, machine learning, extracorporeal membrane oxygenation, ECMO, postoperative ventilation, intra-aortic balloon pump, continuous renal replacement therapy, CRRT, patient management

## Abstract

**Background:**

Postoperative complications contribute significantly to patient morbidity and mortality. Early prediction of such complications could enable the care team to intervene promptly and improve patient outcomes. Existing surgical risk scores are easy to use but lack accuracy and do not provide individualized risk or guidance for clinical decision-making.

**Objective:**

This study aimed to develop and evaluate machine learning models using the INSPIRE perioperative dataset to predict whether an individual patient will require critical postoperative interventions—ventilatory support, extracorporeal membrane oxygenation (ECMO), an intra-aortic balloon pump (IABP), or continuous renal replacement therapy (CRRT).

**Methods:**

Four artificial neural network models and 4 random forest classifiers were trained and tested using the publicly available INSPIRE dataset (131,000 surgical cases). Preoperative laboratory data, medication use, surgery type, and intraoperative vital signs were used as inputs. The dataset was randomly divided into a 70% training set (91,000 cases) and a 30% test set (38,998 cases), with a temporal split to prevent leakage. Model performance was assessed using accuracy, sensitivity, specificity, positive and negative predictive values, and area under the curve (AUC).

**Results:**

The artificial neural network models achieved high predictive performance for ECMO (accuracy 98.9%; AUC 0.992), ventilatory support (accuracy 97.7%; AUC 0.995), IABP (accuracy 98.0%; AUC 0.992), and CRRT (accuracy 94.9%; AUC 0.983). In the test set of 38,998 patients, the neural network models correctly classified 38,569 cases for ECMO, 38,097 for ventilatory support, 38,230 for IABP, and 37,021 for CRRT, corresponding to accuracies of 98.9%, 97.7%, 98.0%, and 94.9%, respectively. The corresponding random forest classifiers achieved accuracies of 98.0%, 99.0%, 98.2%, and 96.5%, respectively, with similar AUC values. These results indicate that the models can reliably identify patients who will or will not require lifesaving postoperative interventions despite the marked class imbalance. Our work began in April 2023, with the research concept taking shape over the following 1 to 2 months. Model development spanned approximately 3 to 4 months and was followed by 1 month of testing and validation conducted by other computer scientists.

**Conclusions:**

Machine learning models trained and tested on the INSPIRE dataset can provide individualized risk estimates for critical postoperative interventions. By supplying accurate predictions before or during surgery, these models can support proactive perioperative planning (eg, intensive care unit bed allocation and device readiness).

## Introduction

Prevention and early detection of complications are often the best treatments in medicine. This is especially true in surgery, where particularly life-threatening complications can occur acutely. As a result, a significant amount of research has focused on how postoperative complications can be accurately predicted at an earlier period and how proactive management based on this information may improve patient outcomes. Over the past few decades, this has mostly occurred with the development of scores or scales that can potentially predict morbidity and mortality risk. A clear example of this is the Surgical Apgar Score (SAS), which uses estimated blood loss, mean arterial pressure, and heart rate to predict patient morbidity or mortality [1]. While such types of scales are easy to use, they happen to have an overall lower accuracy [2]. After conducting a randomized controlled experiment, Manstein et al [2] found the surgical APGAR’s receiver operating characteristic curve to be only 0.661. The authors concluded that “the ability of the SAS to accurately predict risk of postoperative SC [serious complication] at the patient level was limited.” We believe that this limitation is due to the fact that the biomarkers, laboratory values, and vital signs that are clinically relevant predictors of complications for one patient may differ from those that are most informative for another. Therefore, while a scoring system such as the SAS can likely predict postoperative morbidity or mortality in a large percentage of the patient population, there is always a certain subset of patients in which the scale will fail to predict morbidity or mortality (which is where the scoring system fails). Therefore, there is a need to develop a prediction system that can more accurately predict postoperative morbidity and mortality at the individual level and, consequently, at the population level.

Machine learning (ML) has proved to be very proficient in solving such types of problems. Defined as a study of algorithms designed to learn from data and generate insights from unseen data, ML can be used to potentially predict complications based on data such as laboratory results, vital signs, and medication information. Many models that can successfully predict postoperative morbidity (such as postoperative kidney injury [3] or delirium [4]) and mortality have been developed based on this concept. However, although they may be useful for a clinician in predicting a future diagnosis, such models do not provide insights on patient management. By limiting a model’s training to just diagnosis, the utility of the model is also thereby constrained. Knowledge that a patient may develop postoperative kidney injury may encourage a surgeon to be more careful and vigilant during and before the surgery, but planning postoperative management would still be very difficult (especially in time-sensitive situations).

In this work, we aimed to expand upon the current state of the art by creating a proof-of-concept model that encourages proactivity by not only successfully predicting postoperative complications but also providing the physician with clinical decision support to best manage the patient if or when complications occur. We achieve this by creating a set of models that may predict whether a patient will require a certain treatment in the postoperative setting, such as a lifesaving medical device (eg, a ventilator). This can also serve as a proxy for certain management decisions (eg, whether a patient should be transferred to the intensive care unit [ICU] instead of the postanesthesia care unit postoperatively). We then evaluated the model in a dataset comprising 131,000 surgical cases to evaluate the accuracy and robustness of the system.

## Methods

### Overview

At its core, the proposed system attempts to predict whether a patient will require the use of lifesaving devices or services in the postoperative period. The overall architecture of the model and design decisions regarding engineering of the model are discussed below.

The engineered model is an artificial neural network composed of 4 fully connected (dense) layers with SoftMax activation, followed by a final sigmoid activation layer. This 4-layer architecture yielded the highest performance across accuracy, precision, and area under the curve (AUC) metrics. The neural network and activation function was decided upon based on hyperparameter optimization and data type (continuous and dichotomous data). Preoperative laboratory data, medication data, and surgery type served as the input to the model, along with intraoperative vital signs. To clarify, our model uses similar input variables as existing risk scores but uses a novel ML architecture and tuning. The model was developed to predict various different binary labels: extracorporeal membrane oxygenation (ECMO), mechanical ventilation, continuous renal replacement therapy, and intra-aortic balloon pump (IABP). These 4 parameters were chosen due to 3 specific reasons. First, medical devices often require the most amount of time (eg, compared to a medication delivery) to find and set up, especially if their use is not expected. Consequently, the utility of the model is most significantly improved in this setting. A similar rationale applies to predicting postoperative ICU admission; if hospital staff can anticipate the need for ICU care after surgery, appropriate preparations can be made in advance, such as reserving a bed. Note that while we do not model for postoperative ICU admissions (due to dataset constraints), all of the devices that serve as labels in this work require an ICU level of care. Second, the medical devices involved in this model can often dramatically improve a patient’s outcome if used in a timely manner, hence enhancing the utility of the model. Finally, the treatment can serve as a proxy for the patient’s diagnosis. For example, a patient requiring a ventilator or ECMO postoperatively indicates some risk of respiratory failure after surgery. This can potentially enhance a physician’s ability to prevent a complication from happening in the first place. Note that the model is designed to theoretically predict use of other potentially lifesaving devices as well. However, as this is a proof-of-concept study, the scope was limited to the devices described above due to dataset constraints.

The model was trained (defined as the process of feeding data into the model to enable pattern recognition) and evaluated using a dataset named INSPIRE [5], which comprises data from 131,000 patients undergoing surgery from various surgical specialties. Specifically, vital signs from the operating room, ward, and ICU; laboratory results from 6 months before and after discharge; and medication data and general patient characteristics (such as age, sex, and diagnosis) were included. Additionally, complications such as hospital or ICU length of stay, in-hospital death, and use of certain lifesaving medical devices were also included in the dataset. These input data were encoded with a numerical format (male=0.0, female=1.0, etc). Continuous variables were included and not encoded. Patients who required lifesaving medical devices preoperatively were excluded. Missing data regarding a patient were also excluded from the final dataset. In total, 1002 patients were excluded based on the abovementioned exclusion criteria. The postoperative observation period during which the interventions were considered was 30 days before the operation. The dataset was used due to the high patient volume and the presence of postoperative treatment (which matches our specific use case) data. At the same time, inclusion of data from numerous surgical specialties increases the robustness of the model. Specific demographic and operation data from the dataset are shown in Table 1.

**Table 1.** Summary of patient demographics and clinical characteristics (N=131,000).

Characteristic	Values
Age at operation (years), median (IQR)	60 (45‐70)
ASA-PS^a^ classification 1 or 2, n (%)	115,280 (88)
ASA-PS classification >2, n (%)	15,720 (12)
Emergency operations, n (%)	13,100 (10)
Department—general surgery, n (%)	34,715 (26.5)
Department—orthopedic surgery, n (%)	17,423 (13.3)
ICU^b^ admissions, n (%)	14,971 (11.4)
In-hospital mortality, n (%)	1581 (1.2)
ECMO^c^ use (perioperative), n (%)	166 (0.17)
IABP^d^ use (perioperative), n (%)	180 (0.18)
CRRT^e^ use (perioperative), n (%)	855 (0.86)

a ASA-PS: American Society of Anesthesiologists Physical Status.

b ICU: intensive care unit.

c ECMO: extracorporeal membrane oxygenation.

d IABP: intra-aortic balloon pump.

e CRRT: continuous renal replacement therapy.

It is important to note that 1 model will not be used to predict all 4 labels as the labels are not mutually exclusive. Instead, 4 models are generated (each with the same hyperparameters), with a different binary label (rather than a single model with multiple classes). Therefore, when making a prediction, all 4 models will be run to predict the 4 different management possibilities.

The dataset was split into training and test sets in a 70:30 ratio (91,000 to 38,998 patients, respectively) and used a binary cross-entropy loss function due to the binary nature of output labels. All models were trained and evaluated exclusively on the INSPIRE dataset; no local hospital data were used in this study. This ensures that the reported results arise solely from open-source perioperative data and that there was no inadvertent leakage of information from our institution’s clinical records. A temporal split was done to prevent confounding between preoperative and postoperative data. The model was run for 100 epochs, with a batch size of 1000 and a learning rate of 0.0001. To mitigate the severe class imbalance for each intervention label, we applied random oversampling of the positive class in the training data only. Specifically, minority (positive) cases were sampled with replacement until the number of positives matched the number of negatives (1:1). No oversampling or class weighting was applied to the validation or test split; all performance metrics were computed on the original, imbalanced test set. This procedure improved minority class recall while preserving real-world prevalence during evaluation. While it is also possible to decrease the “0” labeled cases to equal the number of cases with the “1” label, this decision was abandoned due to the fact that model accuracy will decrease substantially (due to lack of sample size). After repeated trials, this hyperparameter configuration led to the best learning result. A random forest classification model (which classifies data using an ensemble of decision trees and can help explain model decisions) was also used to better understand model characteristics, such as variable importance (through a Shapley additive explanation [SHAP] analysis), and the decision-making criteria used by the model.

### Ethical Considerations

This study used only the publicly available INSPIRE dataset and involved a secondary analysis of deidentified data. No local hospital data were used, no direct participant contact occurred, and no new informed consent was required for this analysis.

## Results

Model accuracy for both the binary classification model and the random forest model across the 4 features is shown in Table 2. We chose accuracy as our primary metric to ensure that the model is clinically viable. AUC analysis was done to determine the relationship between the false positive and true positive rate. Other methods of clinical validation are also included, such as sensitivity, specificity, negative predictive value, and positive predictive value.

**Table 2.** Model accuracy and area under the curve (AUC) analysis (N=38,998).

Metric	ECMO^a^	Ventilation	IABP^b^	CRRT^c^
Binary classification accuracy, n (%)	38,569 (98.90)	38,097 (97.69)	38,230 (98.03)	37,021 (94.93)
Random forest accuracy, n (%)	38,218 (98.00)	38,608 (99.00)	38,296 (98.20)	37,614 (96.45)
Sensitivity (recall)	0.99	0.99	0.99	0.97
Specificity	0.99	0.98	0.99	0.97
Positive predictive value	0.99	0.98	0.99	0.97
Negative predictive value	0.99	0.99	0.99	0.97
AUC	1.00	0.99	0.99	0.98

a ECMO: extracorporeal membrane oxygenation.

b IABP: intra-aortic balloon pump.

c CRRT: continuous renal replacement therapy.

For each model, we also examined absolute counts of correct vs incorrect classifications. In the test set of 38,998 patients, the artificial neural network models correctly classified 38,569 cases for ECMO (429 misclassifications), 38,097 for ventilatory support (901 misclassifications), 38,230 for IABP (768 misclassifications), and 37,021 for continuous renal replacement therapy (1977 misclassifications). Presenting both percentages and counts facilitates understanding of the clinical significance of the reported accuracies.

While it is difficult to compare the above metrics with the current state of the art (due to slightly different labels, input features, etc), it is clear that the model is able to predict each of the 4 features shown above with a high accuracy based on both evaluation methods. As explained earlier, risk for false negative test results is high in this use case due to the rare likelihood that a patient requires the use of postoperative lifesaving medical devices. While random oversampling was done prior to training to prevent this complication, there are limitations to this technique. Therefore, AUC analysis was done to evaluate both the specificity and sensitivity of the model. This analysis plots the false positive rate against the true positive rate and determines how well a model can distinguish between classes. As shown in Table 2, it is clear that the AUC is substantially above 0.5 (which would indicate that the model is making an entirely random choice or “guessing”), indicating that the models properly identify true positive and true negative results. Given the pronounced class imbalance (rare positive events), the near-perfect AUCs (0.98‐1.00) should be interpreted alongside sensitivity, specificity, positive predictive value or negative predictive value, and absolute correct or incorrect counts.

Further analysis through a random forest classification model reveals insights into the model fit and the features important for the model’s decisions.

**Figure 1.**
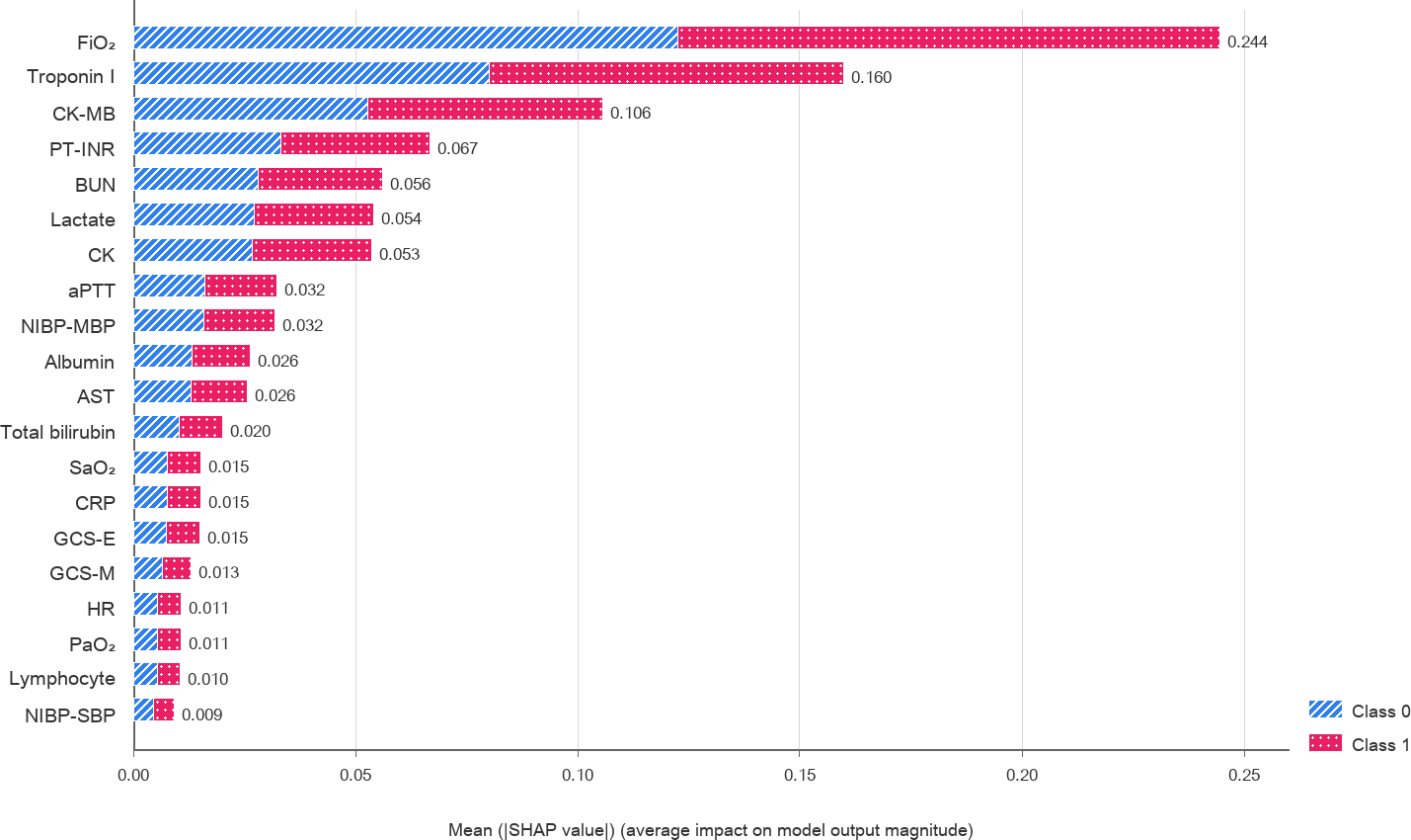
Shapley additive explanation (SHAP) graph of the extracorporeal membrane oxygenation machine learning model. aPTT: activated partial thromboplastin time; AST: aspartate aminotransferase; BUN: blood urea nitrogen; CK: creatine kinase; CK-MB: creatine kinase myocardial band; CRP: C-reactive protein; FiO₂: fraction of inspired oxygen; GCS-E: Glasgow Coma Scale eye response; GCS-M: Glasgow Coma Scale motor response; HR: heart rate; NIBP-MBP: noninvasive blood pressure mean blood pressure; NIBP-SBP: noninvasive blood pressure systolic blood pressure; PaO₂: partial pressure of arterial oxygen; PT-INR: prothrombin time international normalized ratio; SaO₂: arterial oxygen saturation.

Figure 1 presents a SHAP graph (a chart that demonstrates how much a certain variable contributes to the prediction of the ML model) for the ECMO ML model. Analysis of the chart reveals 2 important points. First, the most important variables used by the model are clinically relevant. The fraction of inspired oxygen (FiO₂) feature played a significant role in the decision-making process of the model, while the other features are deemed less important by the model. Indeed, current literature confirms the importance of FiO₂ in ECMO indications. In a review paper by Rabah et al [6], the authors list 8 indications for ECMO device use. Of this list, the specific FiO₂ pressure serves as 3 of these indications. Given that the mean accuracy increases when FiO₂ is added to the feature set, this is correctly reflected in the ML model. This is an important result because it reveals that the decisions made by the model are due to physiologically relevant reasons. This finding is further supported by prior studies showing that lower arterial partial pressure of oxygen to FiO₂ ratios are strongly predictive of ECMO need and correlate with poorer outcomes in critically ill patients [7].

Second, although certain features are not as important as others, they still play a role in the complication prediction for a subset of the patient population. We can see this in the SHAP analysis of the ventilator ML model.

**Figure 2.**
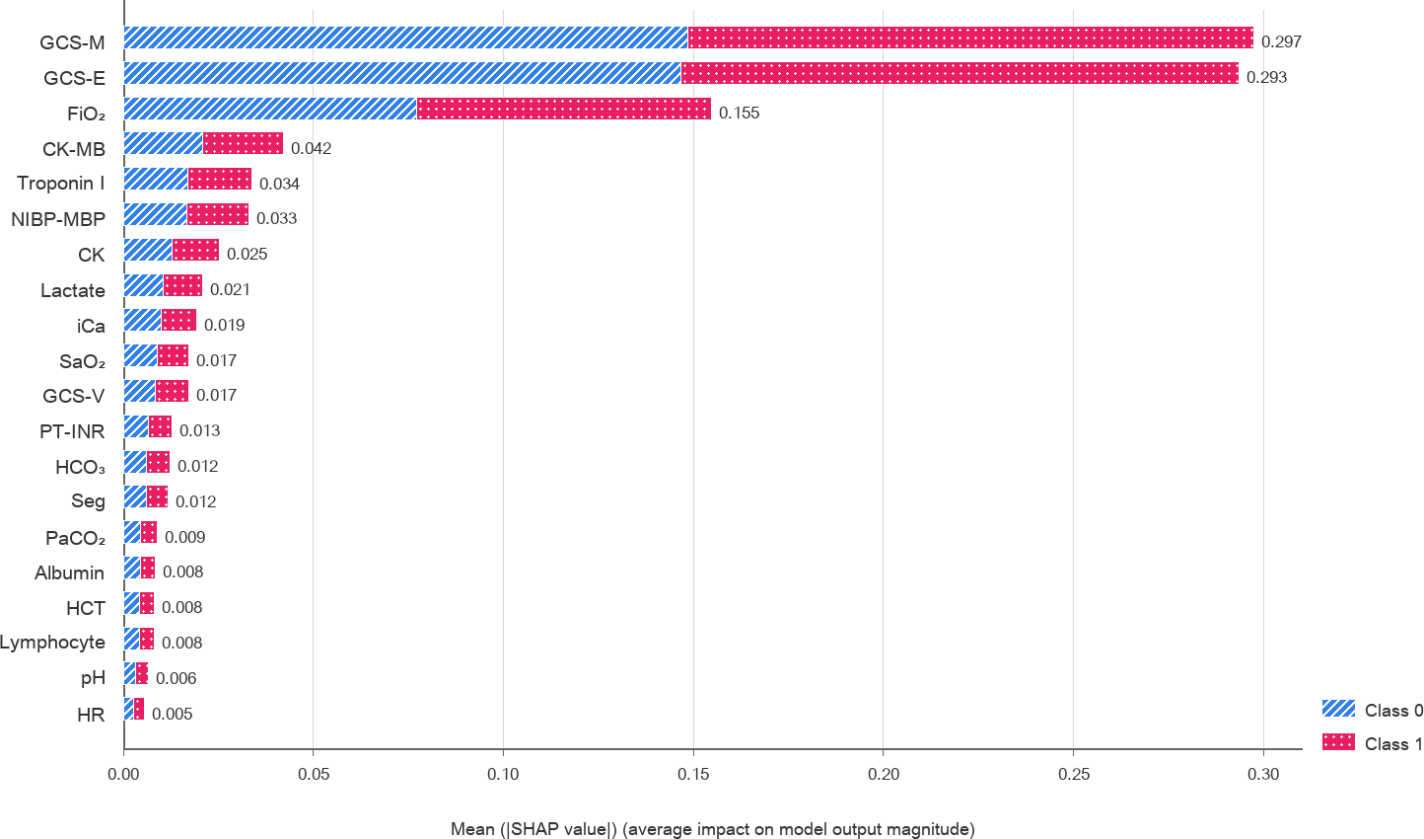
Shapley additive explanation (SHAP) graph of the ventilator machine learning model. CK: creatine kinase; CK-MB: creatine kinase myocardial band; FiO₂: fraction of inspired oxygen; GCS-E: Glasgow Coma Scale eye response; GCS-M: Glasgow Coma Scale motor response; GCS-V: Glasgow Coma Scale verbal response; HCO₃: bicarbonate; HCT: hematocrit; HR: heart rate; iCa: ionized calcium; NIBP-MBP: noninvasive blood pressure mean blood pressure; PaCO₂: partial pressure of arterial carbon dioxide; pH: potential of hydrogen; PT-INR: prothrombin time international normalized ratio; SaO₂: arterial oxygen saturation; Seg: segmented neutrophils.

**Figure 3.**
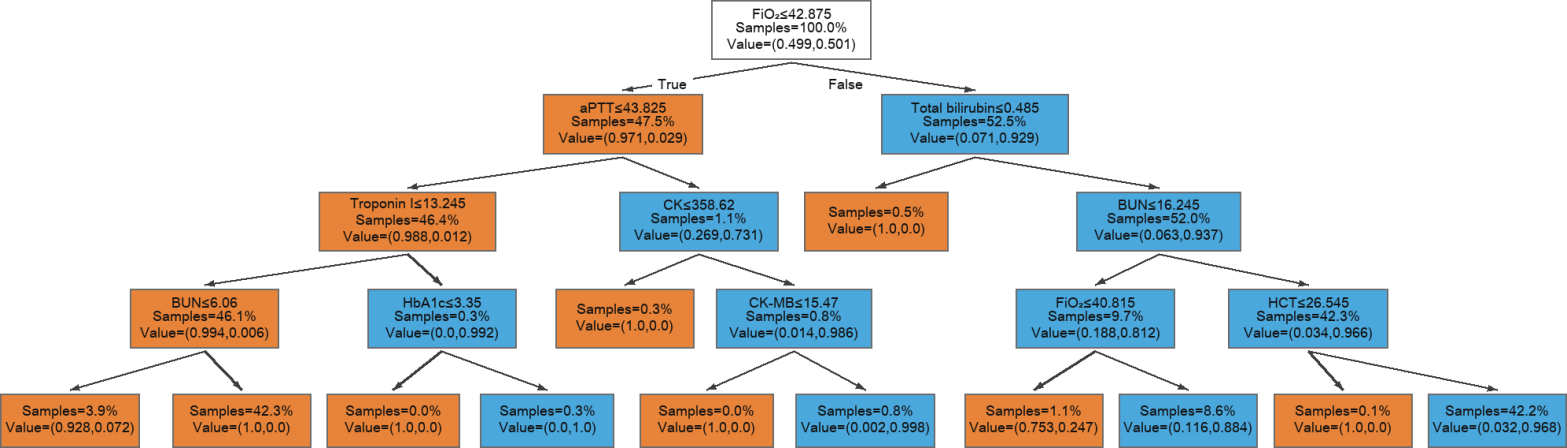
Decision tree graph for the intra-aortic balloon pump model. aptt/aPTT: activated partial thromboplastin time; bun: blood urea nitrogen; ck: creatine kinase; ckmb/CK-MB: creatine kinase-MB, also called creatine kinase myocardial band; fio2/FiO2: fraction of inspired oxygen; hba1c/HbA1c: hemoglobin A1c/glycated hemoglobin; hct: hematocrit; total_bilirubin: total bilirubin; troponin_i: troponin I.

Figure 2 shows that outside of the “gcs_m,” “gcs_e,” and “fio2” variables, accuracy increases only slightly when added into the feature set. This indicates that a certain feature is important in predicting complications in a small subset of the population (rather than the overall patient population), which supports the need for risk analysis at a patient-specific level. If one decides to predict a patient’s complication risk based on 4 or 5 of the most important features, this individual will run the risk of creating a false prediction for a certain group of patients. This can be confirmed by the IABP ML model decision tree shown in Figure 3 (refer to Multimedia Appendix 1 for the expanded version).

The balanced pattern of the tree (which is similar to the trees seen in the other 4 models) indicates that no group of features explains complication risk in an entire patient population. For example, in the last row of the chart, though a certain group of features explains 42.2% (n=54,860) and 42.3% (n=54,990) of the population, there is a certain percentage of patients whose complications (or lack of complications) are not explained by those features. This confirms the need for risk assessment at a patient level rather than at a population level. The alternative reasoning is that the model is overfitting, but we believe that this is unlikely due to 2 reasons. First, the model shows high accuracy and AUC on the test data subset, which the model will have not seen before. Second, the random forest classification was limited to a maximum of 4 layers, which prevents pure memorization of the entire patient population. This 4-layer architecture also yielded the highest performance while preventing overfitting based on the evaluation metrics used above.

Project work began in April 2023, with the research concept taking shape over the following 1 to 2 months. Model development spanned approximately 3 to 4 months and was followed by 1 month of testing and validation conducted by other computer scientists.

## Discussion

The main findings of this study are that it is possible to use ML models to predict whether lifesaving medical treatments would be necessary in postoperative care. In this section, we will discuss the clinical and research benefits of our work and how it can be further improved upon.

Despite significant advances in surgical technique and technology, postoperative mortality still accounts for 0.5% of elective surgical procedures (varying by procedure type and urgency). As a result, a significant amount of research and development has been done to reduce this mortality after a patient’s surgery. With an understanding of an individual’s risk of complications, clinicians can identify complications earlier, better plan postoperative care, or take steps to prevent complications from occurring. Currently, health care guidelines encourage the use of various scales and scores to predict risk of morbidity and mortality (such as the SAS) [2]. While these scores may be efficacious at a population level, they do not predict postoperative mortality at an individual level—an aspect that is often necessary in improving patient outcomes. This is confirmed by the random forest model in the Results section. Moreover, while ML models have been developed to predict postoperative morbidity, we believe that models that improve physician proactivity can better improve patient outcomes.

As a result, we have developed a model that aims to provide physicians with insight on whether a lifesaving medical device is necessary in the postoperative setting. The treatment serves as a proxy for the patient’s diagnosis (eg, a ventilator used in the postoperative setting indicates potential respiratory failure). This provides 2 ideal benefits: better postoperative management of a patient in a potentially critical condition and better preoperative decision-making that can potentially prevent the problem in question. Given the time-sensitive situation of many of these postoperative complications, the practical aspects of patient management, such as alerting the necessary ICU staff beforehand or reserving a lifesaving medical device (if applicable in certain hospital settings), can also be facilitated through this model. As shown in the Results section, the model has a very high accuracy and correctly differentiates true negatives and true positives despite a significantly unbalanced dataset. The random forest classification model reveals a balanced decision tree with an importance graph that reflects current literature.

In practice, such predictions could be integrated into perioperative dashboards to flag high-risk cases before surgery, prompting anesthesiology and ICU teams to prepare necessary equipment and staff. Postoperatively, continuous data streaming from monitors could update risk in real time, allowing automated alerts when a patient’s likelihood of device intervention increases. As this can likely happen in real time through EHR integration, the need for apps or calculators to calculate patient risk scores is also bypassed.

Beyond technical integration, it is essential to demonstrate that the models perform well on the actual patient population. As our proof-of-concept study was built exclusively on the INSPIRE dataset, no data from our own hospital were used for training or testing. We are therefore preparing a retrospective validation study using electronic health record data from our institution. This real-world evaluation will allow us to compare model outputs with actual postoperative interventions, assess generalizability across different demographics and surgical practices, and identify whether recalibration or retraining is necessary. This step is critical for ensuring patient safety before deploying the tool as part of routine perioperative workflows.

Further development is necessary before attempting to use these models clinically. Primarily, we acknowledge that demographic data and laboratory data vary significantly from country to country and even from hospital to hospital, which can potentially change the relevance and real-world accuracy of the system. However, the aim of this study is to develop a proof-of-concept system that can be further retrained and fine-tuned for use in a specific hospital (based on patient data from that specific hospital). This allows for a prediction that is highly accurate for a specific patient population that the hospital serves. Therefore, while we have used a dataset from a university hospital to develop the baseline model, we plan to develop a pipeline that allows ML models to become integrated in other hospital systems, regardless of changes in demographics. Second, as mentioned earlier, there is a very high prevalence of “negative” labels, where the patient did not require the use of these medical device services. Therefore, in a real-world setting, false negatives can occur at a much higher rate. While the positive labels were oversampled to prevent false negative predictions, overfitting of the model is still possible. While evidence of this was not seen in the current state of the work, a different dataset may reveal different results.

Therefore, it is important to test this model in a clinical study to determine both the changes in efficacy and the true clinical relevance of the model. Second, the model should be extended to more predictor variables. While we selected 4 variables for this proof-of-concept study, additional variables can be added as necessary by the physician. By addressing these limitations, we aim to deploy this model in both elective and emergent settings and develop a tool that not only predicts clinical problems but also potentially supports management solutions.

## Supplementary material

10.2196/65327 Multimedia Appendix 1 Expanded decision tree.

## Abbreviations

AUC: area under the curve
CRRT: continuous renal replacement therapy
ECMO: extracorporeal membrane oxygenation
FiO₂: fraction of inspired oxygen
IABP: intra-aortic balloon pump
ICU: intensive care unit
ML: machine learning
SAS: Surgical Apgar Score
SHAP: Shapley additive explanation
