# Supplementary figures and images for: Machine Learning for Predicting Critical Postoperative Interventions: Proof-of-Concept Study Using the INSPIRE Dataset
Source: JMIR Perioper Med. 2026 Jul 28;9:e65327. doi: 10.2196/65327 (PMC13412138; doi:10.2196/65327)

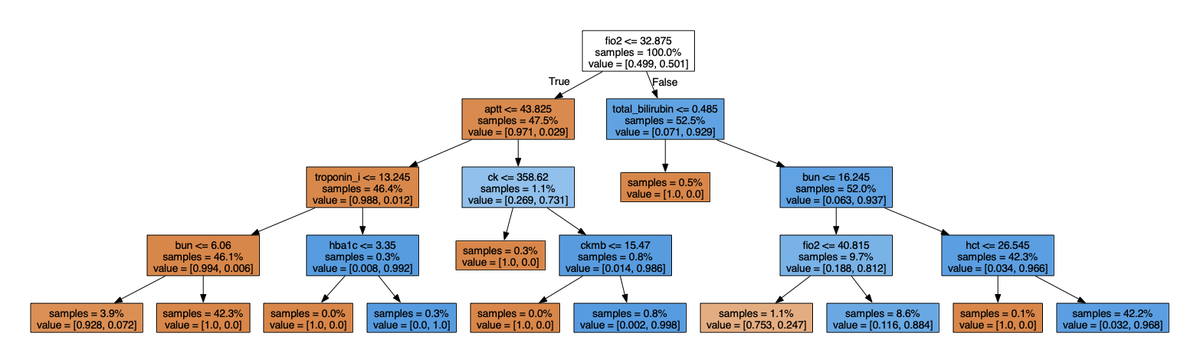

Supplement: Multimedia Appendix 1 [file periop-v9-e65327-s001.png]
